# Gefitinib in Combination with Weekly Docetaxel in Patients with Metastatic Breast Cancer Caused Unexpected Toxicity: Results from a Randomized Phase II Clinical Trial

**DOI:** 10.5402/2012/176789

**Published:** 2012-05-14

**Authors:** Olav Engebraaten, Hege Edvardsen, Erik Løkkevik, Bjørn Naume, Vessela Kristensen, Lars Ottestad, Vasanti Natarajan

**Affiliations:** ^1^Department of Oncology, Oslo University Hospital, 0424 Oslo, Norway; ^2^Faculty of Medicine, University of Oslo, Oslo, Norway; ^3^Institute for Cancer Research, Oslo University Hospital, 0424 Oslo, Norway; ^4^AstraZeneca, R&D, Mereside Alderley Park, Macclesfield, UK

## Abstract

In patients with metastatic breast cancer, taxane treatment demonstrates activity but is not curative. Targeted treatment modalities are therefore necessary in order to improve outcomes in this group. A randomized placebo-controlled phase II trial was initiated to evaluate effect and toxicity of gefitinib (250 mg QD) and docetaxel 35 mg/m^2^ (six of seven weeks) (NCT 00319618). The inclusion of 66 patients was planned. The study was closed due to treatment-related toxicity. Of the 18 included patients, seven (of which three received gefitinib) were withdrawn from the study due to toxicity. Of the nine patients receiving gefitinib and chemotherapy, one achieved a partial response and four stable disease. In the chemotherapy of nine patients, four had a partial response and four stable disease. The breast cancer patients in this study were genotyped using a panel of 14 single-nucleotide polymorphisms (SNPs), previously found associated with docetaxel clearance in a cohort of lung cancer patients. We were unable to identify genes related to toxicity in this study. Nevertheless, toxicity was aggravated by the addition of the tyrosine kinase inhibitor. In conclusion, despite adequately tolerated as monotherapy, combination regimens should be carefully considered for overlapping adverse events in order to avoid increased treatment-related toxicity.

## 1. Introduction

Adjuvant systemic treatment with chemotherapy has improved the outcome for patients with breast cancer [1]. However, in patients with metastatic disease, chemotherapy may prolong survival, but the disease almost always remains incurable, and new therapeutic strategies are therefore needed.

Genetic aberrations and specific molecular pathways that drive the growth and progression of tumors may pave the way for targeted therapies, as in the case of the epidermal growth factor receptors. The amplification of the Her2/neu oncogene with c-erbB2 overexpression in a subset of breast cancer patients is an example of this [2–4]. Breast cancers also overexpress the epidermal growth factor receptor (erbB1), and the presence of this growth factor receptor has been linked to a higher proliferative potential and a worsened prognosis and resistance to hormonal therapy [5]. Despite this, monotherapy inhibiting the EGF (epidermal growth factor—erbB1) receptor with erlotinib or gefitinib in unselected breast cancer patients has been disappointing, without clinical efficacy [6, 7]. Although inhibition of EGFr and downstream MAPK signaling has been demonstrated, this has not translated into a clinical response [8]. However, enhanced efficacy of chemotherapy when combined with EGFr inhibition has been described in an experimental setting [9] and such treatment may even result in an antiangiogenic effect [10]. Treatment with gefitinib in combination with docetaxel was tested in two single-arm, phase II studies with chemotherapy administered every third week [11, 12], shortly after the initiation of our study. The response rate in these studies was relatively high (54% and 39%, resp.). However, the absence of a comparator arm makes it difficult to evaluate these response data [13, 14]. The combination of chemotherapy and tyrosine kinase inhibitors as treatment of NSCLC has been studied with no apparent increase in efficacy in several phase III trials [15–19]. However, chemotherapy in combination with lapatinib (an erbB-2 tyrosine kinase inhibitor) has been highly effective in breast cancer [20, 21], but not without associated toxicity. The combination of the tyrosine kinase inhibitior and chemotherapy has been shown to result in side effects, in particular from the gastrointestinal tract. Toxicity with the docetaxel regimen given every third week is significant, and a better tolerated weekly dosing regimen has consequently been introduced [22–24]. This trial was therefore designed as a randomized phase II study to investigate the potential efficacy and tolerability of gefitinib (an oral EGFR tyrosine kinase inhibitor) in combination with weekly administrated docetaxel.

## 2. Patients and Methods

### 2.1. Patient Selection, Inclusion and Exclusion Criteria, and Randomization

The study was planned for the inclusion of 66 patients with metastatic breast cancer in a placebo-controlled double-blind randomized phase II clinical trial. To be eligible for the study, patients had to be 18 years or older, with a histopathological diagnosis of mammary carcinoma, measurable disease according to the RECIST criteria, and a life expectancy of at least 3 months. The patients signed an informed consent form, had an adequate health status (WHO (World Health Organization) performance status 0–2) with the absence of significant comorbidity (lung or cardiac disease, or previous diagnosis of malignant disease other than basal cell carcinoma), a left ventricular ejection fraction of at least 50% and adequate laboratory values (absolute neutrophil count (ANC) >1.5 × 10^9^/liter, total platelet count ≥100 × 10^9^/liter, serum bilirubin ≤1.0 upper limit of reference range (ULRR), serum creatinine ≤1.5 times the ULRR, alanine amino transferase (ALT) or aspartate amino transferase (AST) ≤2.5 times the ULRR if no demonstrable liver metastases or ≤5 times the ULRR in the presence of liver metastases). If alkaline phosphatase (ALP) was >2.5 times ULRR, the alanine amino transferase (ALT) and/or aspartate amino transferase (AST) had to be ≤1.5 times the ULRR.

Prior to inclusion, patients were required not to have persistent adverse events (common toxicity criteria (CTC) grade 3 or more) from other anticancer treatments and should not have received other treatment modalities. Previous treatment with taxanes was not allowed unless given as a part of an adjuvant treatment regimen more than 1 year before study inclusion. Prior treatment with one antracycline-containing regimen and/or endocrine therapy for metastatic breast cancer was allowed.

A randomization scheme was prepared by the Biostatistics Group, AstraZeneca, and the patients allocated a randomization code strictly sequentially from the pharmacy when entering the study. All information on study drug resided at the pharmacy, including codebreak envelopes to be used in case of medical emergency.

The study was approved by the institutional protocol review board, the regional ethics committee, the Norwegian Medicines Agency and was carried out in accordance with the Declaration of Helsinki, International Conference on Harmony/Good Clinical practice and AstraZeneca's policy on Bioethics. The study was registered in the http://www.ClinicalTrials.gov/ database with the identifier NCT00319618. 

### 2.2. Treatment Plan

The patients were randomized to receive gefitinib 250 mg or a matched placebo tablet once daily during participation in the study. In each seven-week treatment cycle, the patients were given docetaxel every week for six weeks, followed by a one-week treatment rest. Docetaxel was administered at a dose of 35 mg/m^2^, after premedication with dexamethasone given as an IV infusion 30 minutes before administration of chemotherapy. The patients received 8 mg dexamethasone prior to the first and second infusion of docetaxel, and the dose was thereafter reduced to 4 mg dexamethasone. The patients were assessed weekly for adverse events, WHO performance status, and vital signs. Tumor evaluation was performed after each seven-week cycle, and the patients continued on study medication until disease progression or the appearance of unacceptable toxicity.

### 2.3. The Outcome Variables, Evaluation of Tumor Response, and Toxicity Assessment

The primary endpoint of the study was objective tumor response (complete response (CR) or partial response (PR)) according to RECIST criteria in the two treatment groups. The secondary endpoints were time to progression and duration of response. All evaluations were performed according to the RECIST criteria, and all symptoms of toxicity were graded according to the National Cancer Institute common toxicity criteria (CTC version 2.0). The baseline evaluations were performed within 21 days of the inclusion and randomization, and the patients were evaluated after every (7-week) cycle. Patients with skin rash were allowed dose interruptions in the study medication (gefitinib or placebo) for up to 14 days, and supportive therapy such as antibiotics, steroid creams, and antihistamines were administered. In patients with diarrhoea CTC grade 3 or 4, and/or a reduction in ANC, the study medication (gefitinib or placebo) was interrupted until recovery or up to 14 days. Patients unable to restart study medication after this period were taken off the study.

Before the start of a new cycle, the patients were required to have adequate health status and laboratory values (as described in the inclusion and exclusion criteria in Section 2.1). Otherwise, the treatment was delayed until recovery. Patients experiencing treatment delays were retreated after recovery with docetaxel 30 mg/m^2^, except for patients with neutropenia lasting less than 7 days. These patients were retreated at the same dose.

Patients experiencing persistent emesis CTC grade 4, diarrhoea CTC grade ≥3 or peripheral neuropathy grade 2 were retreated with docetaxel at 30 mg/m^2^ upon recovery. Patients with more severe side effects or persistent toxicity despite dose reductions were withdrawn from the study. Patients suspected to have interstitial lung disease (ILD) were immediately taken off the study medication. If ILD was confirmed, the patient was withdrawn from the study.

### 2.4. Hypothesis, Statistical Analysis, and Patient Number Calculations

To warrant further evaluation, the response rate for the gefitinib plus docetaxel treatment arm must be at least 5% greater than the placebo arm. It is hypothesized that gefitinib may increase the objective response rate by 20% over docetaxel alone, an objective response rate for docetaxel of 35% versus 55% for gefitinib plus docetaxel. Assuming the hypothesis is true, the goal of the trial is to have a probability of 0.90 of satisfying both criteria. This study design will require 66 patients (33 per arm) [25].

### 2.5. SNP Analysis

A panel of 14 SNPs in 12 genes (see supplementary Table  1 in Supplementary Material available online at doi:10.5402/2012/176789) previously found to be significantly associated with the clearance of docetaxel in a group of non-small-cell lung cancer (NSCLC) patients were genotyped in this sample set [26]. The SNPs were originally part of a panel of 1030 SNPs genotyped in 193 cancer patients, including the NSCLC patients. The biological justification for our SNP selection in the original panel is thoroughly described in [27] but can in brief be described as SNPs in genes of relevance for the metabolism of reactive oxygen species (ROS) or the response to ROS. The SNPs were genotyped using the 7900HTFast Real-Time PCR System (Applied Biosystems, Foster City, CA), using the standard assay conditions for the Applied Biosystems assay (assay ID given in supplementary Table  1). One of the SNPs could not be genotyped due to failure of the assay design—probably due to large repeat areas in the DNA sequence around the SNP. The DNA genotyped was isolated from either blood or tumor tissue using the QIAamp DNA Mini Kit (Qiagen, Hilden, Germany) and applying the recommended protocol for FFPE (formalin fixed paraffin embedded) tissues.

### 2.6. Statistical Analysis—SNP Studies

The association between individual SNPs and the clinical endpoints was analysed using standard chi-square tests included in SPSS v15.0. *P*-values are two sided and not adjusted for multiple testing. To assess the combinatorial effect of multiple SNPs, leave-one-out cross-validation (LOOCV) analysis implemented in Bioclassifier [28] was utilized. For the LOOCV analysis the clinical parameters needed to be grouped into two groups. This was done, in advance, as described here: treatment response (partial response (PR) and stable disease (SD) versus progressive disease (PD)); WHO score (independent of time point: WHO 0 and 1 versus WHO 2 and WHO 3). The clinical end-point toxicity was already defined as a two-category variable (±toxic response).

No bias for the SNPs studied with regards to tissue type genotyped (blood or tumor) or treatment administered existed for the SNPs except rs1078985 and rs2230949, where one of the alleles was less frequent in the tumors suggesting LOH in the locus (*P*-value 0.048 and 0.041, resp.). No bias was found with regards to treatment regimen or clinical end-points (toxic reaction, WHO score, and treatment response—PR, SD, or PD).

## 3. Results

### 3.1. Patient Demography and Previous Treatment

A total of 18 patients were included in this study. Nine patients were randomized to receive gefitinib 250 mg QD and nine to receive a matched placebo preparation. All patients started treatment with docetaxel 35 mg/m^2^ given as weekly infusions for 6 weeks, followed by a one week without treatment, before the start of a new cycle.

The patient demography was similar in the gefitinib and placebo group, with respect to age and height/weight (see Table 1). Only one patient in each treatment group had received previous chemotherapy for metastatic disease, whereas six patients in the placebo group and three patients in the gefitinib group had received endocrine therapy for metastatic disease. The performance status at study inclusion did not differ, as shown in Table 1.

### 3.2. Study Treatment and Study Discontinuation

The patients randomized to the combined treatment group received a median of 40 doses of gefitinib tablets (range 16–130), whereas the median number of received doses for the control patients was 84 doses of placebo tablets (range 11–161). Fifty-four infusions of docetaxel were planned in each group of patients in each seven-week treatment cycle. However, the patients randomized to receive gefitinib were given 46, 21, and 5 infusions in the first, second, and third treatment cycle, respectively. The corresponding numbers for the patients in the placebo arm were 50, 40, and 25. Only one patient in the study, randomized to the placebo arm, was started on cycle four, but did not finish this treatment cycle. The patients in the combined treatment group received a median of 7.1 weeks of chemotherapy, while the patients treated with chemotherapy alone were given such therapy for 18 weeks (median).

Three patients in the gefitinib-treated group were taken off the study due to treatment-related toxicity, and the treatment for two of these patients was terminated in the first treatment cycle. In the placebo-treated group, four patients were discontinued due to toxicity, one in the first treatment cycle. Correspondingly, six and five patients, receiving gefitinib or placebo in combination with docetaxel, respectively, were taken off the study medication due to objective progression of the disease.

Due to the toxicity seen while on treatment, the study was discontinued after accrual of 18 of the planned 66 patients in the study.

### 3.3. Clinical Response and Time to Tumor Progression

One patient treated with gefitinib and four patients in the placebo-treated group experienced a partial response on the therapy given. All the responses were confirmed and lasted for more than one treatment cycle. The time to tumor progression was median 93 and 131 days (intention-to-treat population), in the gefitinib and the placebo-treated group, respectively. Significance testing was not possible due to the small number of patients included.

### 3.4. Toxicity and Adverse Events

The most commonly encountered adverse events (seen in more than 50% of the patients in either treatment group) are listed in Table 2. Seven patients in each treatment group experienced CTC grade 3 adverse events. One patient in the placebo-treated group had a CTC grade 4 neutropenia during the treatment. Six gefitinib-treated, but only two placebo-treated, patients experienced a grade 3 fatigue. Other than fatigue, gastrointestinal disorders (diarrhoea, nausea, vomiting, and stomatitis) were the most frequent causes of grade 3 adverse events and were seen in three placebo- and four gefitinib-treated patients. Grade 3 dehydration and electrolyte disturbances were seen in four patients treated with gefitinib, but not in patients treated with placebo. One patient treated with gefitinib was found to have a deep vein thrombosis, and one placebo-treated patient had a subclavian vein thrombosis, both CTC grade 3 events.

The general status of the patients differed between the groups during the first treatment cycle, reflected in the WHO performance status reported. After four weeks in the study, none of the patients treated with gefitinib and docetaxel remained in the WHO grade 0 category, and at the end of the treatment cycle, four patients had a WHO grade 2 performance status. The patients treated with placebo and docetaxel had a better performance status with three patients on WHO grade 0 at 4 weeks and none experiencing a grade 2 (or worse) performance status at the end of the treatment cycle.

Five patients treated with gefitinib and docetaxel and three patients treated with placebo and docetaxel were reported with serious adverse events (SAEs—see Table 3). The majority of the serious adverse events were linked to the occurrence of diarrhoea, reduced oral fluid and food intake and fluid loss in four patients treated with gefitinib and docetaxel and two patients treated with placebo and docetaxel. All these patients needed supportive hospitalization. For one patient in each treatment group, multiple SAEs were reported, with two events for the patient treated with gefitinib and three events for the placebo-treated patient.

The SAEs in the patients treated with gefitinib and docetaxel, experiencing catheter sepsis and pneumonia, were not considered related to the study medication. This was also the case for the patient with subclavian vein thrombosis, who received placebo and docetaxel.

### 3.5. SNP Analysis

Due to the relatively large proportion of patients with adverse events in both the gefitinib- and placebo-treated groups, we wanted to investigate if the toxicity seen could be related to genotypic determinants of docetaxel metabolism. For this study, we used a panel of 13 SNPs in 12 genes, identified in a previous study where these SNPs were tested in a serie of 24 non-small-cell lung cancer patients treated with docetaxel [26]. These SNPs were associated with the clearance of docetaxel in these patients and were found in genes related to the metabolism of or response to reactive oxygen species.

#### 3.5.1. Association between Individual SNPs and Toxicity

Analyzing the genotype distribution of the 13 studied SNPs separately against the clinical end-points revealed two associations with borderline significance: (1) the association between the SNP rs701992 in *NDUFB4* (NADH dehydrogenase (ubiquinone) 1 beta subcomplex, 4) and the WHO score at 0 weeks of treatment (*P*-value 0.055) and (2) the association between the SNP rs1341164 in *CYP2C8* (Cytochrome P4502C8) and the WHO score at 8 weeks of treatment (*P*-value 0.044). In both cases there is a higher frequency of the mutated allele in the groups with higher scores for the clinical end-points. The genotype distribution of these SNPs associated with the WHO performance status at before treatment (SNP rs701992) and after 8 weeks of therapy (SNP rs1341164) can be found in supplementary Table 2.

#### 3.5.2. Combinatorial Impact of the Genotyped SNPs on Response Using LOOCV Analysis

To assess the collective impact of multiple SNPs on the clinical end-points we performed a leave-one-out cross-validation analysis [28]. For the clinical end-point response we combined the patients in two groups: either partial response or stable disease (PR/SD) or progressive disease (PD). Our panel of SNPs correctly classified the response in these two groups in 83.3% of the samples (*n* = 15, Table 4). More close analysis at the LOOCV (leave one out cross validation analysis) result points to the rs2228001 in *XPC* (xeroderma pigmentosum complementation group C) gene contributing mostly to this predictive potential. To further investigate this association with an alternative statistical method, a chi-square analysis in the same two-response groups was performed, and a higher frequency of the T allele in the group with progressive disease was observed (*P* = 0.057, Table 5).

## 4. Discussion

The aim of this study was to investigate the feasibility and activity of combining the oral tyrosine kinase inhibitor gefitinib with docetaxel. The tolerability of chemotherapy has generally been improved by the introduction of weekly based dosing regimens, as demonstrated for both paclitaxel and docetaxel with no reduction in efficacy (for review see [29]). Gefitinib dosed daily at 250 mg is very well tolerated and has also been extensively studied in combination with different chemotherapeutic regimens in many disease settings [30]. However, despite an excellent safety profile of these agents when given alone, the addition of other therapies may change the tolerability of the chemotherapy treatment. In our study, the majority of adverse and serious adverse events were related to fluid balance, fluid intake, and dehydration and other gastrointestinal side effects, as described previously for weekly treatment regimens [22, 29]. Only one patient experienced neutropenia as a serious adverse event. This is in contrast to the two published studies on the combined treatment with gefitinib and docetaxel given every third week [11, 12]. In these studies, neutropenia associated with chemotherapy administration was the most frequent adverse event. Severity of neutropenia was grade 3 or 4, affecting 43 and 49% of the patients, respectively. In one of the studies, the patients were allowed to continue in the study until disease progression or other causes for withdrawal, and 30% of the patients discontinued the treatment due to toxicity [12]. In the other study by Ciardiello et al., the patients were allowed treatment for up to 6 cycles (36 weeks) of combined therapy; thereafter gefitinib monotherapy was administered to patients experiencing a clinical response [11]. The median number of chemotherapy cycles in these studies was 5.6 and 5.2, respectively, corresponding to 16.8- and 15.5-weeks treatment duration. In our study, the patients in the placebo group were given chemotherapy for 18 weeks (median), comparable to the previously mentioned studies, while the patients treated with gefitinib received chemotherapy for 7.1 weeks (median). Despite this, patients who discontinued due to toxicity were present in each group (four in the placebo-treated group at 3.4, 13.4, 18, and 22 weeks, and three in the gefitinib group at 2, 5, and 19.7 weeks). The reason for the short treatment period in the gefitinib treatment group is therefore both due to patients experiencing toxicity and progression of the disease, and not only caused by the combined treatment with gefitinib and docetaxel. The reported WHO performance status was also worsened in patients treated with the chemotherapy/gefitinib combination. Whether this is due to the treatment is uncertain, as the time to tumor progression was shorter in the gefitinib/docetaxel treated patients, and a clinical deterioration may have resulted in a worsened performance status.

However, the fact that the patients in the gefitinib group reporting fatigue grade 3 and dehydration and electrolyte disturbances (also grade 3) were only found in the gefitinib treatment group (four patients) may indicate an increased toxicity of combined treatment with docetaxel and gefitinib. This is also supported by the serious adverse events observed (see Table 3). However, the number of patients is small and the results should therefore be interpreted with caution. The increased tolerability of weekly dosed chemotherapy is mainly associated with a reduction of hematologic side effects, but other nonhematologic adverse events may become more important when combination therapy is introduced, as seen in our study. Treatment with gefitinib seemed to be less well tolerated when given in combination with docetaxel dosed weekly (our study) compared to the every third-week dosing regimens [11, 12].

Patients treated with gefitinib had a shorter time to tumor progression, and only one of these patients experienced a partial response, compared to four patients treated with weekly docetaxel alone. Due to the small number of patients treated, it is not possible to evaluate the efficacy and activity of the gefitinib/docetaxel combination. Although some studies have reported efficacy of the combined treatment with gefitinib and docetaxel [11, 12], the role of gefitinib in obtaining these responses is not clear, and further clinical studies are needed to address this question.

In a study investigating the influence of 550 SNPs on docetaxel clearance in 24 patients with NSCLC treated at our institution, a number of SNPs were found to be associated with docetaxel clearance [26]. To study whether these results could predict the response or toxicity seen in our patients, we genotyped the cohort for the presence of these SNPs. Two SNP rs701992 in *NDUFB4* and rs1341164 in *CYP2C8* were found weakly associated with WHO score at 0 and 8 weeks of treatment, respectively (see supplementary Table  2). One SNP, rs2228001, in *XPC* was found at higher frequency of the T allele in the group with progressive disease. The results must be interpreted with caution, due to the limited number of patients included into the present study, and do not give an answer to the question of the predictive value of such a panel of SNPs. The *XPC* gene, giving the predictive potential, encodes a key protein in the nucleotide excision repair (NER) pathway, and a recent study has linked the presence of T in this allele (Ala499Val) to a reduced DNA damage and treatment response *in vitro* [31]. Other studies have identified the XPC gene to be changed after treatment [32], and that genetic variation may influence the risk of recurrence after treatment for malignant neoplasms [33], but little is known about the effect of such genetic variants on docetaxel and/or gefitinib sensitivity, and further studies are needed to clarify this.

 Association between survival and metabolism genotype has been shown in patients treated with anthracyclines and cyclophosphamide [34, 35]. Docetaxel is metabolized by the cytochrome P450 system, and in particular the CYP3A enzymes [36]. Drugs like docetaxel and gefitinib, which share the same metabolic pathway, may influence the breakdown of each other *in vivo*, increasing the drug concentrations. This could affect both the toxicity and effect of the drugs [37]. However, associations between genetic variants of the CYP3A enzymes and the effect of docetaxel have not, to our knowledge, been found, although pharmacogenomic studies have been published investigating the possible influence of genetic variants on the docetaxel metabolism [38–40]. The genetic variants of the CYP3A enzymes have not been investigated in this study, and a relationship between such variants and the observed toxicity cannot be excluded. However, the number of patients in our study is small, and larger studies with more patients are needed for obtaining reliable conclusions about such a relationship.

In conclusion, the weekly dosing regimen of docetaxel used in our study did not improve the treatment tolerability, as expected from other publications and our clinical experience. Although the majority of the toxicity most likely is due to the chemotherapy treatment alone, treatment with gefitinib may have contributed and aggravated the toxicity. Some of the patients treated with the combination regimen experienced dehydration and electrolyte disturbances not seen in patients treated with chemotherapy alone and not seen in patients treated with the same drug in other dosing regimens. Gefitinib has been successfully combined with other therapies (hormonal therapy and chemotherapy), without severe combined toxicity [30]. Nevertheless, increased gastrointestinal toxicities and rash have been observed. In the planning of clinical studies with apparently well-tolerated targeted therapies in combination with chemotherapy, the possibility of overlapping adverse events should be carefully considered to avoid increased toxicity of the treatment regimen.

## Supplementary Material

Supplementary Table 1 contains information on genomic position, suggested function, associated gene and assay ID for the studied SNPs.

## Figures and Tables

**Table 1:** Patient demography at screening.

	Docetaxel alone (*n* = 9)	Gefitinib + docetaxel (*n* = 9)
Age (range; years)	53.1 (35–69)	56.3 (37–67)
Weight/height	70.4 kg/164 cm	70.4 kg/164 cm
Performance status		
WHO grade 0	5	4
WHO grade 1	3	4
WHO grade 2	1	1
Previous therapy for metastatic disease		
None	1	5
Endocrine therapy	6	3
Antracycline	1	1

**Table 2:** Common adverse events during treatment.

Event	Docetaxel alone (*n*)	Gefitinib + docetaxel (*n*)
Diarrhoea	8	8
Nausea	8	5
Vomiting	5	3
Fatigue	8	9
Rash	4	6

*n*: number of patients experiencing adverse event.

**Table 3:** Serious adverse events.

Event	Docetaxel alone (*n* = 3)	Gefitinib + docetaxel (*n* = 5)
Dehydration	0	3
Fatigue	0	1
Stomatitis	0	1
Diarrhoea	3	2
Hypokalemia	0	1
Anorexia	1	0
Vomiting	1	0
Neutropenia	0	1
Pneumonia	0	1
Infection	0	1
Catheter sepsis	0	1
Chest pain	0	1
Subclavian vein thrombosis	1	0
Vertigo	1	0

*n*: number of patients.

**Table 4:** Classification results from the LOOCV analysis*.

Patient ID	Response	LOOCV analysis	
		True class	Classified as
1	2	SD + PR	SD + PR
2	3	PD	PD
3	1	SD + PR	SD + PR
4	2	SD + PR	SD + PR
5	2	SD + PR	SD + PR
6	3	PD	PD
7	2	SD + PR	SD + PR
8	1	SD + PR	SD + PR
9	1	SD + PR	SD + PR
10	2	SD + PR	SD + PR
11	2	SD + PR	SD + PR
12	3	PD	SD + PR
13	1	SD + PR	SD + PR
14	3	PD	SD + PR
15	1	SD + PR	SD + PR
16	3	PD	PD
17	2	SD + PR	SD + PR
18	2	SD + PR	PD

*Patient IDs given in bold are correctly classified with regards to response group in the analysis.

**Table 5:** Genotype distribution of rs2228001 in the treatment response categories partial response/stable disease versus progressive disease*.

Clinical end-point	Genotype distribution			
	GG	GT	TT	*P* value
PR + SD	5 (38.5)	7 (53.8)	1 (7.7)	
PD	1 (20.0)	1 (20.0)	3 (60.0)	0.057

*Number in brackets indicate percentages within each clinical level.
