# Supplementary material for: Gefitinib in Combination with Weekly Docetaxel in Patients with Metastatic Breast Cancer Caused Unexpected Toxicity: Results from a Randomized Phase II Clinical Trial
Source: ISRN Oncol. 2012 May 14;2012:176789. doi: 10.5402/2012/176789 (PMC3361199; doi:10.5402/2012/176789)
Supplement: Supplementary file 1 — Supplementary Table 1 contains information on genomic position, suggested function, associated gene and assay ID for the studied SNPs. [file 176789.f1.pdf]

Supplementary Table 1. Genotyped SNPs with info on gene association, chromosomal localisation, function of the SNP and the assay id from Applied Biosystems

Gene	SNP-ID	Function	Chr	ABI Assay ID
CYP2C8	rs1341164	Intron	10	C___7537656_10
EPHX1	rs2234922	Coding exon	1	C__11638783_30
GSTA4	rs1032419	3'UTR	6	C___8910269_10
IGF2	rs2230949	Exon	11	C__15853775_20
IL1R2	rs2236930	Intron	2	C__15955373_20
MAPK7	rs1042855	3'UTR	17	C___7443511_20
NDUFB4	rs701992	3'UTR	3	C___8761891_10
PRDX4	rs795491	Intron	X	*
TGFBR2	rs1078985	Intron	3	C____972343_10
TGFBR3	rs2253316	Intron	1	C__16066451_10
TPMT	rs2842951	Intron	6	C____396305_10
	rs7886	Promoter	6	C__12091548_10
XPC	rs2607737	Intron	3	C___1661682_10
	rs2228001	3'UTR	3	C____234284_1_

*Manufactures were unable to design an assay for rs795491
The SNP is located in a repeat rich area of the genome which may explain the diffucutlies.

Supplementary Table2. Genotype distribution of the SNPs found significantly associated with clinical end-points. Number in brackets indicate percentages within each clinical level.

Clinical end-point		Genotype distribution			
A.		rs1341164			
WHO score (T=8)		AA	AT	TT	p-value
	0	1 (50.0)	0	1 (50.0)	
	1	8 (88.9)	1 (11.1)	0	
	2	2 (50.0)	2 (50.0)	0	0,044
B.		rs701992			
WHO score (T=0)		AA	AT	TT	p-value
	0	5 (55.6)	4 (44.4)	0	
	1	3 (42.9)	4 (57.1)	0	
	2	1 (50.0)	0	1 (50.0)	0,055
